# Nasopharyngeal and Oropharyngeal Swabs, And/Or Serology for SARS COVID-19: What Are We Looking For?

**DOI:** 10.3390/ijerph17093289

**Published:** 2020-05-08

**Authors:** Alessandro Sanduzzi, Stefano Sanduzzi Zamparelli

**Affiliations:** 1 Section of Respiratory Disease, Department of Clinical Medicine and Surgery, Monaldi Hospital, Federico II University, 80138 Naples, Italy; stefanosanduzzi@gmail.com; 2 Staff of The United Nations Educational, Scientific and Cultural Organization (UNESCO) Chair on Health Education and Sustainable Development, Federico II University, 80131 Naples, Italy

**Keywords:** SARS, COVID-19, serology, swab, RT-PCR

## Abstract

Governments and clinicians that were fully involved in the dramatic SARS-CoV-2 outbreak during the last few weeks in Italy (and more or less all over the world) are fiercely debating the use of methods for screening this viral infection. Thus, all countries are employing a lot of resources in order to test more and more subjects. For this purpose, there are different strategies, based on either direct or indirect tests. Among the first category, the main assays used for SARS-CoV-2 are based on a real-time reverse transcriptase polymerase chain reaction (RT-PCR). Such tests can be performed on nasopharyngeal and oropharyngeal swabs for the categories of those with symptoms and those potentially exposed. In order to integrate the molecular assays in the diagnosis of SARS-CoV-2, a wide range of serology immunoassays (IAs) have also been developed. If we want to identify “immune” people in order to let them to come back to work, serology is the best (and probably the only) approach.

On March the 16, 2020, Tedros Adhanom Ghebreyesus, WHO General Director, concluding his speech about SARS-Cov-2 to the United Nations Assembly pronounced the famous sentence: “test, test, test”.

Governments and clinicians that where fully involved in the dramatic SARS-CoV-2 outbreak during the last few weeks in Italy, (and more or less all over the world) are fiercely debating the use of methods for screening this viral infection. Thus, all countries are employing a lot of resources in order to test more and more subjects. For this purpose, there are different possible strategies, based on either direct or indirect tests:

## 1. Direct Tests

The main assays used for SARS-CoV-2 are based on a real-time reverse transcriptase polymerase chain reaction (RT-PCR) that needs a few hours to give an answer. Most molecular tests have been approved by the United States Food and Drug Administration (FDA) under emergency use authorization (EUA) and are Conformité Européenne (CE) marked [1,2].

Such tests can be performed on nasopharyngeal and oropharyngeal swabs in symptomatic people (fever, dry cough, asthenia). Another strategy is to test all health care workers and individuals in the potentially exposed category (policemen, military); nasopharyngeal and oropharyngeal swabs could also be used for those in close contact with SARS-CoV-2-positive people or for people who live in close and crowded settings (e.g., nursing homes). Theoretically, nasopharyngeal and oropharyngeal swabs could be performed for the whole population (some Italian regions that are trying to start this program are wondering if such an approach could be logistically achievable and economically sustainable).

Nevertheless, we must keep in mind that swabs results can show a certain degree of variability for the following reasons:

(a) the test’s efficiency depends on the adequacy of specimen it is not infrequent to have false negative results (the swab must be collected deeply to obtain adequate material);
(b) one or more negative results do not rule out the possibility of SARS-CoV-2 infection, because a number of different factors could lead to a negative result in an infected subject, including:
  - sampling mistakes (the specimen could be collected too late or too early in the infection course);
  - shipment mistakes (the specimen has not been appropriately handled and shipped);
  - technical reasons related to the test (e.g., virus mutation or PCR inhibition that could interfere with the result of the test [3]).
(c) in case of recent exposure to infection, a subject previously negative could become positive: therefore, this kind of test is not useful for screening, but rather in the case of clinical suspect and it can be repeated in the case of a new suspect. Even if in recent clinical practice there is the trend to repeat swab, the correct meaning of serial results is still to define, underlying that the use of serial sampling seems to be to monitor clearance. One possible explanation of resulting variability in serial specimens in the same subject could be the different viral load, though, at the moment nobody knows which viral load cut-off is necessary to define infectiousness. Besides, it is very difficult to understand the real meaning of the persistence of a positive swab in patients that are clinically recovered, a trait that is not infrequent to observe in medical practice.

Further research is needed to determine effectiveness and reliability of repeated sampling.

## 2. Indirect Test

In order to integrate the molecular assays in the diagnosis of SARS-CoV-2, a wide range of serology immunoassays (IAs) have also been developed. Among the most frequently used IAs, there are automated chemiluminescent IA (CLIA), manual ELISA, and rapid lateral flow IA (LFIA), which detect the immunoglobulin M (IgM) and immunoglobulin G (IgG) produced in people infected by SARS-CoV-2 [4]. Ou says that while cross-reactivity in antibody binding to the spike protein is common, cross-neutralization of the live viruses is rare, indicating the presence of a non-neutralizing antibody response to conserved epitopes in the spike. Whether these non-neutralizing antibody responses will lead to antibody-dependent disease enhancement needs to be addressed in the future [5].

Quite recently, the role of serology seems to be increasing: in Italy, some regions like Tuscany, Emilia-Romagna, and Piemonte are beginning to create an epidemiological survey on at risk populations (health care workers, nursing home operators, etc.), following the indications of Germany, South Korea, Singapore, United Kingdom. The aim of this plan is to estimate the virus’s prevalence in the general population.

Maybe it is necessary to take a breath and consider what we are doing and what we are looking for:

- Serology is the best (and probably the only) approach if we want to identify people likely to be immune, or at least not contagious, in order to let them to come back to work. It should be useful to see in vitro neutralization tests with patient serum in comparison with ELISA tests of the same serum samples in order to see if and how well these two correlate.

Let’s start to underline a couple of considerations:

- The median incubation period of such a virus is estimated to be 5.1 days.
- IgM anti SARS-CoV-2 becomes detectable just 7–8 days after onset of symptoms.

Thus, the evaluation and the titration of specific IgM and IgG antibodies can clarify the timing of infection, and such a result could be obtained both cases of symptomatic and asymptomatic people. At the moment the main role of serological tests is to give a retrospective assessment of the attack rate or the extent of an outbreak [6].

In this sense, only IgG positive IgM negative people, with two consecutively negative SARS-CoV-2 PCR assays and resolution of clinical symptoms, could realistically be considered cured (or at least not contagious), even if there are some reports showing reinfection, although sporadic, with SARS-CoV-2 (among the most reliable hypothesis, there could be new viral strains, mutations or immunity failure [7]). Thus, the effective immune protection of IgG antibodies against reinfection with the same virus is not yet clear, or otherwise, low titers of such Abs could not be efficient for a whole and permanent protection. In fact, the levels of SARS-CoV-2 antibodies vary widely in patients after recovery, and although infrequently, IgG antibodies could be undetectable in some patients: how these patients recovered without the help of antibodies and whether they were at risk of reinfection of SARS-CoV-2 should be further explored [8]. Soon after disease onset in a mild case, Dahllke observed an increased frequency of plasmablasts concomitantly with a strong SARS-CoV-2-specific IgA response, while a case with more severe progression showed a delayed, but eventually a very strong and broad SARS-CoV-2-specific IgA response [9].

- To diagnose the active disease, such a method could not be useful, except if we intercept the onset of IgM positivity. Besides, screening protocols should be adapted to the local situation. For a whole, although schematic, framing, see Table 1.

In conclusion, the performance of such assays needs to be critically evaluated before they are employed alone for the clinical diagnosis of COVID-19, which in clinical practice, continues to be based also on high resolution computed tomography pattern, combined with functional, laboratory, and clinical data, in addition to direct tests.

## Figures and Tables

**Table 1:** Who what, and when to test for COVID-19 diagnosis/screening. From “Laboratory testing for coronavirus disease 2019 (COVID-19) in suspected human cases”, interim guidance, 2 March 2020, WHO, modified [10].

Who	Test	Type of Sample	Timing
Suspected patient	Direct test	Nasopharyngeal and oropharyngeal swabs	Collect on presentation *
Suspected patient	Serology	Serum **	Initial sample in the first week of illness and the second ideally collected 2–4 weeks later
Contact ***	Direct test	Nasopharyngeal and oropharyngeal swabs	Within incubation period of last documented contact
Recovered patient	Serology	Serum **	Optimal timing for convalescent sample needs to be established

*Possibly repeated sampling to monitor clearance; ** for serology testing, once validated and available; *** in health-care center-associated outbreaks or other settings where contacts have symptoms, or where asymptomatic contacts have had high-intensity contact with a COVID-19 case.
